# Alcohol and Cardiac Arrythmias: A Review of the Current Data

**DOI:** 10.31083/j.rcm2404105

**Published:** 2023-04-06

**Authors:** James Kilcoyne, Abdalrahman Assaassa

**Affiliations:** ^1^Saint Mary Physician Group, Comprehensive Cardiology Section, Langhorne, PA 19067, USA; ^2^Saint Mary Medical Center, Internal Medicine Residency, Langhorne, PA 19067, USA

**Keywords:** alcohol, sudden cardiac death, atrial fibrillation, arrythmia

## Abstract

**Background:**

Alcohol is a pervasive substance in the US and the world in general. Cardiac arrythmias, specifically atrial fibrillation, are also a critical health issue. The interplay between alcohol and arrythmia is explored here.

**Methods:**

Original research, editorials and other literature reviews were searched and assessed for candidacy for inclusion and ability to contribute to this article.

**Conclusions:**

Alcohol consumption has a significant interplay with cardiac arrhythmia.

## 1. Introduction

Alcohol consumption is a regular phenomenon among most regions and cultures of the world, although patterns vary. It has been evident that the first alcohol preparation was 8000 BC when humans started building sedentary communities. The earliest proof was through chemical analysis of pottery jars found in Jiahu, North China [1]. In 2016, World Health Organization (WHO) estimated that more than 2.3 billion people worldwide were current drinkers [2]. 83.1% of US citizens are current or former regular consumers of alcohol, whereas in Europe this numbers stands at 76.6% and within the African continent is only 42.6%. Interestingly, those that due consume alcohol on a regular basis take in on average 33–37 g/day. Women tend to abstain from alcohol more and have a lower incidence of binge drinking [3]. Alcohol consumption trends show that related disorders are likely to rise as per capita consumption has increased 2.9% from 2019–2020, with this trend set to continue [4]. More than 3 million people died due to harmful alcohol consumption with almost 19% of all deaths attributable to alcohol consumption worldwide were due to cardiovascular diseases.

In the US, alcohol consumption varies among the subpopulation groups. Moreover, health consequences differ among racial and ethnic groups. While the highest prevalence was reported among white respondents, native Americans and blacks were more affected by alcohol-related health consequences [2].

Although low to moderate alcohol consumption has been associated with lower rates of all-cause mortality, nonfatal acute myocardial infarction and coronary death, multiple studies demonstrate that the relation between alcohol intake and all-cause and cardiovascular mortality has a J-shaped or U-shaped pattern [5].

Alcohol consumption is associated with many side effects in different systems including the cardiovascular system. High levels of alcohol intake found to be associated with an increased risk of hypertension and overall incident hypertension. Compared with no alcohol intake, consumption of >2 drinks daily is associated with an increased risk of all stroke outcomes. In another study, 33% of dilated cardiomyopathy cases were attributed to alcohol cardiomyopathy [6].

Although not fully understood, alcohol has also arrhythmogenic effects through multiple mechanisms including electrophysiological, chemical, and autonomic disturbances. This is explaining the data that shows an increased risk of atrial and ventricular as well as sudden cardiac death.

In this review, we will discuss the literature studying the relationship between alcohol and cardiac arrhythmia including causality, types and the level of alcohol intake and the underlying possible pathophysiological mechanisms.

### Methods

Research and construction of the review article was performed as follows. Literature search on scholarly research search engines were performed utilizing Pubmed, Ovid, Google Scholar. Primary research was prioritized. Keywords such as “Atrial Fibrillation”, “Alcohol”, “Arrythmia”, “Sudden Cardiac Death”, among others were used. This information was reviewed by both authors and both authors contributed significantly to this article.

## 2. Alcohol and Sudden Cardiac Death

Sudden cardiac death is a very dramatic and serious event in a patient’s life. Thankfully statistics in treatment of sudden cardiac death (SCD) are improving, with more patients surviving to hospital discharge on a yearly basis. Victims and survivors of SCD are a heterogenous group of patient, with many factors contributing to their initial event and total survival [7]. As alcohol consumption is such a common social phenomena, investigation into its role in SCD risk has been examined, with ultimately complex findings. In one study among 22,071 male physicians, a total of 141 sudden cardiac deaths were documented over the 12 year monitoring period. Those who consume >5 drinks/day were found to have an increased risk of ventricular arrhythmia and sudden cardiac death. Conversely, 2–4 drinks/week and 5–6 drinks/week were found to decrease the relative risk of SCD by 60% and 79%, respectively. This reflects the U-shaped association between alcohol consumption and SCD [8]. A similar finding was found also among women, with a U-shaped association between light-to-moderate alcohol consumption and an increased risk of SCD. The lowest risk of SCD observed among women who consumed 5.0 to 14.9 g of alcohol daily (approximately 0.5–1 drink per day). Interestingly, the risk among women with the highest bracket of alcohol intake (≥30 g or ~≥2 drinks/day) did not significantly differ from the risk observed among the abstainers. This lack of a risk of SCD at peak alcohol intake is unexplained but suggests some unknown confounding factors [9]. This seemingly protective effect of alcohol on SCD is felt to be related to beneficial effects on mechanics and thrombosis. As mentioned above, the etiology of SCD are varied although ischemic heart disease is felt to be a common cause. In a study in the United Kingdom though, only 54% of deaths due to ischemic heart disease were classified as SCD. in addition to this heavy drinker, in this study classified as >6 drinkers per day, did show increased risk of SCD although did not show higher rates of fatal myocardial infarction. In addition, deaths that did occur from ischemic heart disease were more likely to be sudden in heavy alcohol consumers, suggesting a potential potentiating effect of alcohol in these patients [10]. Alcohol as a chronic risk factor does appear to have an impact on SCD, but the acute impact of alcohol also as been explored. Among victims of SCD, individuals were 3 times more likely to suffer SCD within 2 hours of alcohol consumption than those that did not, with peak time to death of 1.5 hours from alcohol consumption. These individuals often had 3 risk factors for ischemia heart disease. Many factors adjusted the risk of SCD, including physical activity and resting tachycardia, suggesting that alcohol intake was one of many factors leading to SCD [11]. As the cause of SCD can be varied, alcohol influence on it is likely mediated by other, more direct causes. Association between alcohol consumption and SCD due to nonischemic heart disease was observed among the Finnish victims with all deaths happened in Finland between 1998–2017. Of total of 1301 SCD deaths, the blood ethanol level was elevated in 543 (42%) subjects, out of which the blood alcohol level was ≥0.10% in 339 (62%) subjects and ≥0.15% in 252 (46%) subjects. These results were noted more between males compared to females, which follows general consumption trends throughout the world [12].

## 3. Alcohol Consumption and Other Atrial Arrhythmia

Although many studies were done between alcohol consumption and Atrial fibrillation association, less studies applied on the effect of other atrial arrhythmia. Ettinger *et al*. [13] as one of the first who studies the effect of excessive alcohol intake and cardiac arrythmia. Among 24 patients with recent alcohol ingestion, atrial fibrillation in addition to atrial flutter, atrial tachycardia, junctional tachycardia, multiple premature atrial contractions, multiple premature ventricular contractions (PVCs) and ventricular tachycardia were observed [13]. Kupari *et al*. [14] studied 289 patients aged <65 who were admitted for supraventricular tachyarrhythmia. 102 patients have idiopathic arrhythmias with patients who have episodes starting in Saturday and Sunday were most likely to be chronic alcohol drinker. Further analysis showed that the time of arrhythmia onset was correlated with CAGE questionnaire response rather than most recent alcohol use. This study emphasized the strong association between heavy drinkers on weekend and idiopathic supraventricular arrhythmia. This study did though find a relative increase weekend/holiday supraventricular arrhythmias, somewhat cooling the idea of the classic “Holiday Heart” [14].

In MunichBREW study, they prospectively enrolled 3028 voluntary participants in whom smartphone based electrocardiograms (EKGs) were analyzed for cardiac arrhythmia (including sinus tachycardia, sinus arrhythmia, premature atrial/ventricular complexes, atrial fibrillation/flutter) associated with breath alcohol concentration (BAC) measurements. In this study mean age of participants was 34.4 ± 13.3 years with 29% of them being women. Mean BAC was 0.85 ± 0.54 g/kg. Using multivariable adjusted logistic regression, cardiac arrhythmias occurred in 30.5%, almost entirely supraventricular tachycardias. Breath alcohol concentration was significantly associated with cardiac arrhythmias in general (odds ratio (OR) per 1-unit change 1.75, 95% confidence interval (CI) 1.50–2.05; *p *< 0.001), sinus tachycardia showing an odds ratio of ~2 [15].

## 4. Protective Effect of Alcohol on Cardiac Arrythmias

Although many studies illustrate the positive association of alcohol consumption and atrial fibrillation, few studies found protective effects of alcohol. Psaty *et al*. [16] studied the independent risk facors of new onset atrial fibrillation among old adults. They found that high level of alcohol consumption was associated with decrease risk of atrial fibrillation among all patients with or without clinical vascular disease [16]. It is not clear how this factors in to the rest of the literature that establishes a negative effect of alcohol on atrial fibrillation as well as general cardiovascular risk.

### Atrial Fibrillation

Atrial fibrillation is absolutely no stranger to any healthcare practitioner in the US or worldwide. In 2017, 37.5 million people carried the diagnosis of atrial fibrillation with 3.5 million new cases that year, with these numbers expected to increase over time [17]. This is a serious public health issue as atrial fibrillation contributes to multiple consequences such as stroke, affecting 2.5 million Americans per year [18]. The World Health Organization estimates that 6% of global deaths are related to alcohol use, and alcohol related cardiovascular disease is related to 12% mortality [19]. Alcohol consumption is also a worldwide issue that contributes significant to various diseases [20]. Acute alcohol intoxication has classically been associated with so called “Holiday Heart”, with atrial fibrillation developing within 12–36 hours of binge alcohol intoxication [21]. Chronic alcohol consumption is associated with the development of various arrythmias including atrial fibrillation, sinus tachycardia and PVCs [22], with 33% of patients with atrial fibrillation being considered alcoholics. High alcohol consumption is directly associated with an increase in atrial fibrillation, with more moderate consumption being controversial at times [23]. The populations of the ONTARGET and TRANSCEND trials were evaluated for their alcohol consumption. They found that moderate alcohol consumption, 1–14 drinks/week in women and 1–21 drinks/week in men, increased the risk of atrial fibrillation with a hazard ratio of 1.16 compared to low intake, which was <1 drink/week. High alcohol intake and binge drinking, >21 drinks/week, increased risk of atrial fibrillation further, with a hazard ratio of 1.33 compared to low intake. Conversely all-cause mortality was improved with all cause death being 9.9% and 10.6% in the moderate and high intake group compared to 12.55% in the low intake group [23]. The concern over this type of alcohol consumption pattern is that average alcohol consumption can vary according to country, with Norway averaging 3.8 gram per day, equivalent to 2 drinks per week as 12 grams of alcohol was considered a standard drink [24]. In this group, 23% of the patients had 3+ drinks per week [24]. There seems to be an inflection point at 1 drink a day or cumulative drinks per week where atrial fibrillation risk increases [24]. It would seem also that more alcohol is worse given other studies suggesting a hazard ratio of 1.46 for the highest quintile of alcohol consumption, equivalent to 68 grams/day of alcohol [25]. Interestingly studies have found the ranges of alcohol consumption, 1–9 drinks per week, increase atrial fibrillation burden although are acceptable intake patterns per American Heart Association guidelines [26, 27]. Type of alcohol do not seem to matter in regard to beer, wine or spirits, as accounting for these does not change the incidence of atrial fibrillation [25]. Assessment of gender in this matter is not well fleshed out although when women are separated out, no association with alcohol and atrial fibrillation can be found [28]. Further sub-populations that have minimal data are young adults. Pathologic alcohol consumption patterns peak in late adolescence and early adult hood [29]. Han *et al*. [30] looked in young adults aged 20–39 years old. Patients with at least mild consumption of alcohol for 4 years, or higher weekly consumption for shorter time periods, had a 25% higher risk for atrial fibrillation than non-drinkers. This risk did seem to scale with consumption as young adults with >210 g per week of alcohol consumption over 4 years had further risk enhancement of 47% over non-drinkers [30]. Levels of alcohol intake also predict transition from paroxysmal to permanent atrial fibrillation [31]. Alcohol’s relationship with atrial fibrillation is further cemented with the evidence that abstinence from alcohol decreases atrial fibrillation burden in those with atrial fibrillation (AF) and have regular alcohol consumption. These findings were confounded by weight loss that occurred with alcohol abstinence, with weight loss also independently being associated with decreased AF burden [26]. While chronic alcohol consumption has been an intense area of research more recently, the association with alcohol and atrial fibrillation started with the signal of acute intoxication. Ettinger *et al*. [13] studied chronic heavy alcohol consumer with recent alcohol ingestion, admitted to the hospital for atrial fibrillation with rapid ventricular response. They found that the arrythmia resolved once patients achieved abstinence for a period of time [13]. Alcohol withdrawal is also complicated by atrial fibrillation with mortality during withdrawal admission being worse if atrial fibrillation is present [32].

To counter a majority of the data available, there are some indicators of alcohol being protective of atrial fibrillation. UK BioBank participants aged from 49–69 years old showed a J-shaped curve in regard to alcohol consumption. Individuals consuming 1–7 drinks per week actually had less atrial fibrillation than those who had <1 drink per week, with 5 alcohol beverages per week being the nadir of risk of atrial fibrillation [33]. At these lower doses of alcohol exposure, beer/spirits consumption had a linear risk of AF while wine (red and white) appeared to add the protective aspect of alcohol consumption. There has been some postulation that resveratrol in wine is the beneficial component, as animal studies show that it mediates decreased fibrosis and ion-channel regulation, although human study is pending [33].

Alcohol’s direct relationship with atrial fibrillation is fairly clear but tangential effects are also just as important. As has been well established, atrial fibrillation is associated with a risk of stroke. To mitigate this risk, therapeutic anticoagulation is prescribed to appropriate patients [20]. Reddiess *et al*. [34] found that alcohol consumption did not enhance stroke risk in those with atrial fibrillation. The obvious question therefore is what is the interplay between alcohol and risk of anticoagulation. The databases for the Swiss-AF and Ground-Breaking Electroporation-based intervention for Atrial Fibrillation Treatment (BEAT-AF) were assessed for bleeding events as a secondary outcome. They found that non-drinkers, classified as 0 to <1 drinks per day had a comparable bleeding risk to those that drank more than 2 drinks per day [34]. In addition, patients that are prescribed warfarin therapy, as well as score high on alcohol abuse questionaries show a 2-fold higher rate of major bleeding, even when international normalized ratio (INR) is taken into account [35]. Conversely, alcohol consumption does increase risk of thromboembolism or death (Hazard Ratio: 1.33) in males with a heavy alcohol consumption pattern, >27 drinks per week. Women also see an increase in thromboembolism, although with lower consumed of 20+ drinks per week. These effects are durable what accounting for anticoagulant use [36].

While certainly alcohol has an effect on arrythmia, the question remains is it a primary driver of arrythmia or does it predispose to a phenomenon that is already to occur. Shared risk factors such as hypertension and obesity are associated with development of atrial fibrillation as well as being related to increased alcohol consumption [37]. Hypertension is clearly seen in chronic consumers of alcohol, with a statistically linkage starting with 3 drinks a day. There is clearly cross over between previously mentioned mechanisms, such as renin-angiotensin-aldosterone stimulation, vasoreactivity due to intracellular calcium increase and inflammation and oxidative injury. Autonomic activation has been found to be integral in rats, with alcohol stimulating the adrenal glands to increase heart rate, cardiac output and systolic blood pressure [38]. Hypertension, even mild hypertension has been associated with significantly increased left atrial size [39]. This increase in size is associated with decreased chance that sinus rhythm will be able to be maintained and is a strong predictor of development of nonvalvular atrial fibrillation [40]. Similarly obstructive sleep apnea (OSA), especially if left untreated, can lead to increased atrial fibrillation risk and burden. This obstructive sleep apnea promotes arrhythmogenesis through oxidative stress and excess sympathetic activity. Meta-analyses have shown that alcohol consumption increases the risk of OSA by up to 25% [41].

## 5. Electrophysiology and Pathological Consequences of Alcohol

The basis for alcohol induced arrhythmogenesis is primarily based on its effects in the myocardium. In silico models of both ventricular and atrial tissue, alcohol has been shown to alter ion channel function and myocytes currents. Alcohol reduces sodium, calcium and transient-inward potassium channel function. Alcohol also enhances $I_{\text{calcium}}$ and sarcoplasmic reticulum calcium release. This will lead to prolongation of atrial action potential duration. This effect scales up with high concentrations of alcohol in the atria. Ventricular tissue shows no electrophysiologic effect at low doses of alcohol (BAC of 0.04%) although at higher dose which mimic binge drinking (3.64% BAC) action potential duration is prolonged [42]. Binge-level alcohol consumption causes increase in T-type calcium current, pulmonary vein cardiomyocytes being particularly sensitive to this [21]. Calcium handling is critical to atrial fibrillation generation and maintenance through delayed after depolarizations (DAD). Intracellular calcium overload can lead to increased DAD, and with another amplitude, these can cause cell depolarization and firing [43]. These DADs can be the focus of initiation to start reentry. In addition, with atrial tachycardia, calcium influx is minimized through $I_{\text{cal}}$ channel to prevent intracellular calcium toxicity. This reduction in $I_{\text{cal}}$ activity prolongs action potential duration as well as action potential duration (APD) rate dependency, within the atrial myocardium as well as the pulmonary vein cardiomyocyte. This leads to great AF inducibility [21]. Both these actions promote reentry [43]. There does appear to be a balancing effect though within calcium handling. High frequency activity in the cardiac membrane leads to increased refractory state of the sarcoplasmic reticulum’s ryanodine receptor. These actually lead to a down regulation of calcium handling promoting and working against triggered activity generation [18]. In this way, triggered activity of calcium overload is suppressed to some degree.

In atria tissue models, low dose ethanol increased reentry duration and shifted vulnerable window to a shorter stimulated S1S2 interval, leading to a higher vulnerability for stable arrythmia. Higher doses, 10-fold atria dose, was needed to cause this same effect on the ventricle. This effect of increased stable reentry was also seen in long-standing atrial fibrillation models although atrial fibrillation rotors were seen to be more meandering with higher alcohol concentrations, likely promoting further propagation of atrial fibrillation. Heart failure models have also shown similar increased stable reentry with increasing alcohol concentrations [42]. Heart failure also shows altered intercellular coupling, predominantly at higher doses. This is accomplished by prolonged reentry vulnerable periods [42]. Conversely, low dose alcohol led to reduced reentry through Ik1 channel inhibition, though in atrial fibrillation this effect is ameliorated due to atrial fibrillation effect on Ik1 channel. Inflammation is well known to be associated with atrial fibrillation instigation. Transient T2-signal intensity seen 1 day after binge drinking suggests myocardial edema, hyperemia and inflammation with excess alcohol consumption. This inflammation leads to Interluekin-6 down-regulation and therefore conduction slowing through gap junction protein connexins 43 and 40. These gap junction proteins are found predominantly in the atria, linking inflammation to atrial arrhythmias [21].

All the above, as well as other mechanisms, lead to autonomic dysfunction as a complication of alcohol consumption. Increased sinus tachycardia often occurs with acute intoxication. Vagal tone also plays a key role in arrhythmogenesis. In a study of 15 healthy volunteers which were given ethanol infusion. Markers of vagal activity such as periodic repolarization dynamics as well as deceleration capacity were assessed during infusion achieving maximum blood alcohol concentration of 0.5 mg/L, intoxicating doses. Periodic repolarization dynamics was increased and deceleration capacity was decreased showing reduced parasympathetic activity/vagal activity [44].

## 6. Alcohol and Structural Remodeling of the Heart

Another mechanism of arrythmia in atrial fibrillation is fibrosis and scar. Increasing fibrosis is potentially protective in forming reentry in the atrial although interestingly increased the duration and stability of reentrant arrythmias in the ventricular. After myocardial infarction, remodeling of border zone ion channels occurs. Mild to moderate fibrosis is not affected by alcohol but the presence of alcohol reduced reentrant arrythmias in extensive fibrosis [42]. Another mechanism of alcohol pathology is oxidative stress. Increased Nicotinamide adenine dinucleotide+Hydrogen/Nicotinamide adenine dinucleotide (NADH/NAD+) ratio with accumulation of acetaldehyde is a consequence of ethanol metabolism. This decreases intra-cellular antioxidant enzymes levels/activity. This lack of antioxidant protection will lead to myocyte disarray, apoptosis and contractile protein fragmentation. These changes are seen within 2–18 weeks of alcohol consumption. Reactive Oxygen Species (ROS) scavenger administration prevented alcohol-related scarring, apoptosis and cardiac structure alternations [6]. Further evidence of ROS-species linking atrial fibrillation and alcohol was shown through investigation done through Lung-AN Hsu *et al*. [45]. They generated mitochondrial aldehyde dehydrogenase 2 (ALDH2) to assess the effect on alcohol and AF with and without protection from reactive-oxygen species. Test and control mice both showed similar inducibility of AF without alcohol administration. With ethanol administration, ALDH2 knockout mice showed higher 4-hydrozy-ytrans-2-nonenal (4-HNE) levels and greater AF inducibility. These aldehydes being present are also fibrogenic, through the TGF-beta 1 pathway, which was confirmed by ALDH2 knock out mice showing more severe fibrosis after ethanol consumption compared to control [45]. Alcohol intake caused increased angiotensin II and norepinephrine levels [6]. Angiotensin II activates angiotensin II type 1 receptor which stimulates fibroblast proliferation and apoptosis and cardiomyocytes, which contributes to fibrosis of the atria. Therapeutics such as Angiotensin-Converting Enzyme-inhibitors have been shown to prevent this fibrosis of the atrial and reduce associated atrial fibrillation [43].

## 7. Conclusions

While atrial fibrillation is a complex topic, with many contributory factors, alcohol consumption remains an important modifiable risk factor in the US as well as worldwide. Through direct electrophysiologic effects as well as worsening other risk factors for atrial fibrillation, alcohol has a direct and largely negative effect on atrial fibrillation. An important gap in knowledge and study is research of alcohol nd arrythmias other than atrial fibrillation. This is an important topic that would be a crucial direction of study going forward. Overall, their seems to be a significant arrythmogenic risk when it comes to almost any alcohol consumption, although “binge” drinking is a definite risk. Interestingly, level of alcohol consumption noted in this review that can lead to pathology and overt arrythmias actually fall within the acceptable range of alcohol consumption by United States Preventative Task Force guidelines. By 2018 publication, unhealthy alcohol consumption is defined as more than 4 drinks per day for men under 65 years old and more than 3 drinks for women of any age and men over 65 years old [46]. As mentioned above, even alcohol consumption under this limit can cause arrythmia. This can be understandably confusing to clinicians, not to mention patients that are trying to live a “healthy” life. The above information is further support for clinicians counseling patients on healthy lifestyle habits, although discussion of appropriate alcohol consumption takes into account not just the arrhythmogenic risks, but all potential effects.
